# Succession characteristics of phytoplankton functional groups and ecological assessment in a cold spring-type urban lake, China

**DOI:** 10.3389/fmicb.2024.1435078

**Published:** 2024-07-17

**Authors:** Henglun Shen, He Xu, Xinru Zhang, Tianshun Zhu, Wanxiang Jiang, Xin Li

**Affiliations:** ^1^College of Life Sciences, Zaozhuang University, Zaozhuang, China; ^2^Jinan Environmental Research Institute, Jinan, China

**Keywords:** Daming Lake, phytoplankton functional group, Q index, environmental factors, redundancy analysis

## Abstract

Phytoplankton functional groups have been increasingly utilized in elucidating and predicting the response of phytoplankton species to environmental conditions and seasonal succession in various aquatic systems including lakes, rivers and reservoirs. However, it is still unclear whether the trait-based functional classification can be applied to spring-type lakes. To understand the temporal and spatial characteristics of phytoplankton functional groups and their responses to environmental factors in spring-type urban lake in northern China, an investigation was conducted in Daming Lake from May 2020 to September 2021. The findings revealed the identification of 98 phytoplankton taxa belonging to 6 phyla, predominantly being Chlorophyta (39.8%), Bacillariophyta (35.7%) and Cyanophyta (15.3%). The dominant species were *Microcystis* sp., *Merismopedia minima*, *Synedra acus* and *Scenedesmus quadricauda*. These phytoplankton taxa were categorized into 21 functional groups, with 6 dominant functional groups (abbreviated as D, MP, P, J, Lo, and W1). Among them, the functional group D, primarily constituted by *S. acus*, exhibited absolute predominance. The seasonal succession sequence of the dominant functional groups was as follows: D/P/J/MP/ Lo →→ D/P/W1/MP/Lo/J → D/P/J → D/MP → D/P/MP. Throughout the investigation period, the trophic level index (*TLI*) ranged from 39.10 to 71.13, and the Q index was from 1.91 to 2.91, both indicating a medium health state for Daming Lake, which was consistent with the evaluation results of the diversity index. The results of redundancy analysis revealed that the main driving factors of phytoplankton FG biomass and composition were water temperature (WT), total nitrogen (TN), transparency (SD), TN:TP (N:P), redox potential (ORP), chemical oxygen demand (COD_Mn_) and pH. The dominance of the functional group D positively correlated with water temperature, TN, COD_Mn_, pH and N:P but negatively correlated with SD. It was observed that functional groups and the Q index can objectively indicate the seasonal succession of phytoplankton and the water quality status of Daming Lake. Our discoveries have significant implications for the comprehension of the effects of urbanization on phytoplankton dynamics and for enhancing lake management practices to foster sustainable urban development.

## 1 Introduction

Natural processes and escalating anthropogenic activities have propelled the phenomenon of eutrophication in urban aquatic systems, resulting in heightened crises such as ecosystem degradation and diminished biodiversity, posing a serious threat to the vitality of urban aquatic ecosystems (Han et al., 2022; Zhang et al., 2023). Compared to environmental factors, the dynamics of biological communities provide a comprehensive reflection of various environmental changes and more directly and comprehensively indicate the water ecological health status (Qu and Zhou, 2024). Phytoplankton, as important primary producers and the foundation of aquatic food web, are widely recognized as natural bioindicators for detecting alterations in aquatic ecosystems owing to their swift responsiveness to environmental conditions (Shen et al., 2022; Anderson et al., 2024). Taxonomic classification of phytoplankton primarily relies on morphological descriptions, which fail to fully capture their ecological function in the ecosystem (Padisák et al., 2009; Wiltshire et al., 2022). Consequently, numerous studies have utilized functional diversity to represent functional aspects of phytoplankton communities adopting functional groups (FGs), morpho-functional (MFG) and morphology-based functional group (MBFG; Reynolds et al., 2002; Salmaso and Padisák, 2007; Kruk et al., 2010). While each method has its advantages and limitations, the FG method stands as the most comprehensive approach, encompassing morphological characteristics, habitat characteristics, and ecological characteristics of phytoplankton (Zhao et al., 2023). The FG method better elucidates their adaptability to the environmental changes and more accurately reflect the relationship between environmental factors and their ecological niche (Shen et al., 2022; Costa et al., 2023). Additionally, this method has been extensively employed in ecological health assessment and water environment evaluation of lakes, such as Dongting Lake and the Oswego Lake (Grund et al., 2022; Yan et al., 2023), providing direct insights into the seasonal succession characteristics of phytoplankton functional groups in urban lakes.

Ecological researches on urban lake systems encompasses both large lakes like Xuanwu Lake in Nanjing, Simco Lake in Canada, and Okichoby Lake in the United States, as well as small urban lakes such as Xihu Lake in Tongling (Kelly et al., 2017; Ma et al., 2022; Wang et al., 2022; Qu and Zhou, 2024). The urbanization process led to elevated nutrient inputs, particularly nitrogen and phosphorus, resulting in potential eutrophication, Urban lakes are vulnerable ecosystems, sensitive to eutrophication (Florescu et al., 2022; Guo et al., 2023). Physicochemical parameters, such as water temperature, nitrogen, phosphorus, chemical oxygen demand and light also play crucial roles in shaping the phytoplankton community structures in urban lakes (Vascotto et al., 2024; Zheng et al., 2024). Urban lakes generally exhibit higher nitrogen and phosphorus concentrations compared to other type of lakes, with Cyanophyta, Bacillariophyta and Chlorophyta being dominate species throughout the year, displaying relatively high abundance and biomass (Florescu et al., 2022; Guo et al., 2023; Li et al., 2023).

Daming Lake, a cold spring-fed urban lake, primarily received water from surrounding springs and surface runoff channeled through the west moat, suffered serve eutrophication from 1996 to 2004 due to the discharge of excessive nutrients like nitrogen, phosphorus, and organic matter from the densely populated residential and commercial areas nearby (Zheng et al., 2017; Dai et al., 2022). Despite notable improvement in infrastructure and natural and artificial landscape following the expansion projects in 2009, the continuous discharge of high-concentration sewage into the lake, owing to the dense population in old city of Jinan, remains a pressing concern (Dai et al., 2022). In terms of nutrient levels within these bodies of water, it was found that high nitrate content in the spring waters flowing into Daming Lake resulted in an increased total nitrogen concentration averaging about 9.96 mg/L within its incoming rivers, a significantly higher than those found within Daming Lake itself (unpublished data). At present, research on Daming Lake mainly focuses on water quality and heavy metals in sediment, while studies on the functional ecosystem are limited (Zheng et al., 2017; Lu et al., 2019; Dai et al., 2020). Notably, a comparative analysis on stability of phytoplankton functional groups in Daming Lake has yet to be undertaken.

This study investigates the seasonal succession pattern of the phytoplankton community and the environmental factors influencing this succession. The objectives of the study were to pinpoint the key drivers of shifts in the dominant functional groups within phytoplankton communities, assessing the ecosystem health of Daming Lake using diversity indices, ecological state index (Q), and comprehensive trophic level index (TLI) and proposing recommendations for enhancing water quality. These objectives were achieved through analyzing water quality and functional group characteristics of phytoplankton communities during lake restoration. This study also furnishes essential insights into the alterations in phytoplankton composition resulting from changes in aquatic environmental factors during lake restoration, and offers guidance on the selection of functional group classification methodologies for phytoplankton in urban lakes characterized by a spring-type water regime.

## 2 Materials and methods

### 2.1 Investigation area

Daming Lake, located in the center of Ji’nan (36°40′N, 117°01′E), which is a warm temperate continental monsoon climate and known as one of Jinan’s three historical scenic spots. It is a lake formed by the convergence of multiple springs, including Pearl Spring Group, Five Dragon Spring Group, and Baotu Spring, as well as atmospheric precipitation and surface runoff (Ding et al., 2013). Following a dredging operation in 2010, the lake expanded to cover an area of 0.58 km^2^, boasting an average depth of 2.0 m and a water storage capacity averaging 0.83 km^2^ (Fu et al., 2021).

### 2.2 Sample collection and processing

**Figure 1:**
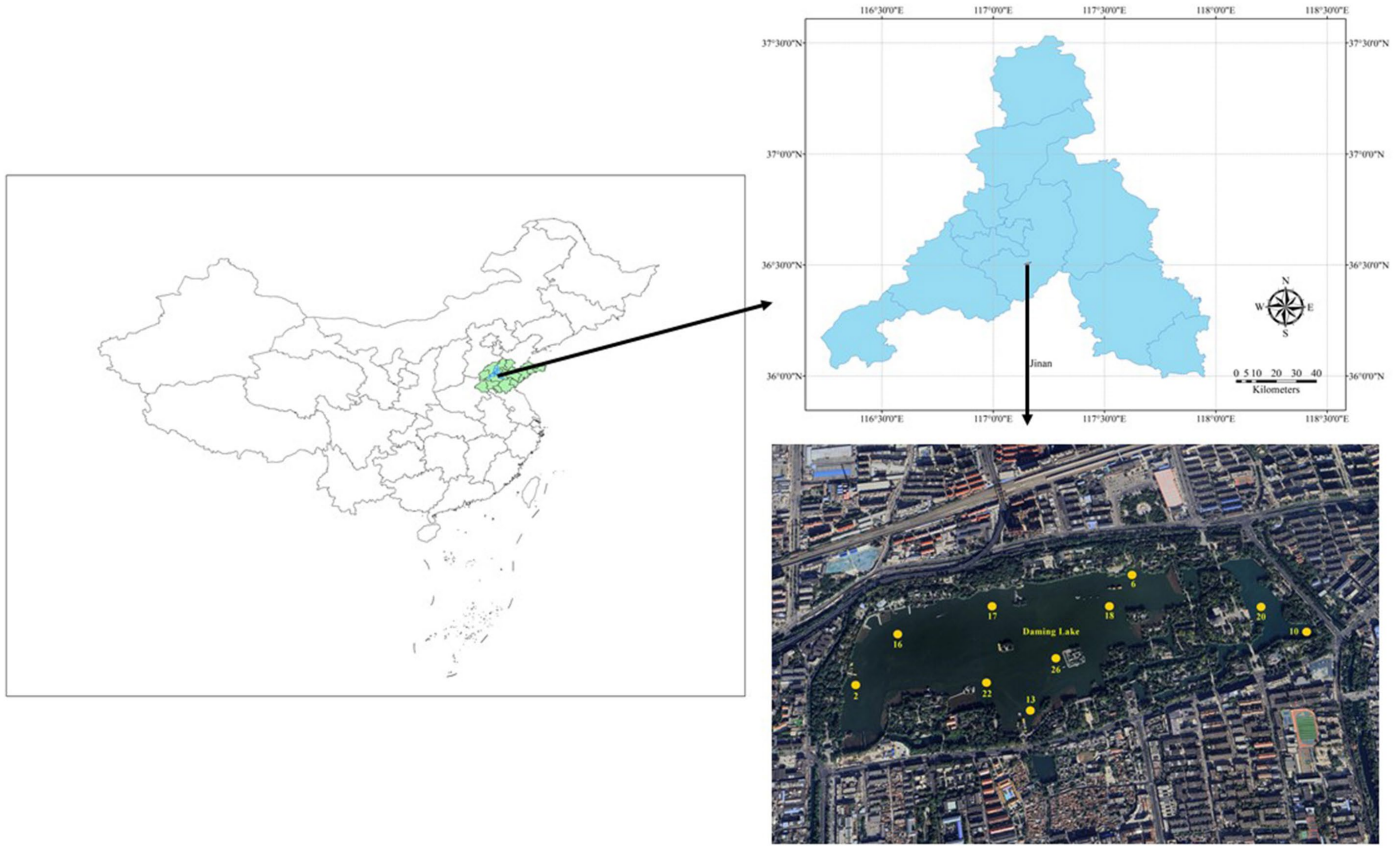
Geographical location of Daming Lake and sampling sites.

Based on the lake area distribution, 10 sampling sites were chosen at Daming Lake, utilizing a handheld global positioning system (GPS) device (Figure 1). DMH02 was situated at the primary inlet, DMH10 at a newly excavated outlet; and DMH13 at the secondary inlet sourced from Pearl Springs (Dai et al., 2020). Samples were collected quarterly from May 2020 to September 2021 to represent seasonal variations in water conditions. In addition, due to construction and repair reasons, DMH10 and DMH22 did not undergo sample collection in 2021. Surface water samples (0–0.5 m) were collected in triplicate using brown glass bottles at each sampling for subsequent analysis.

The *in-situ* measurements included water temperature (WT), dissolved oxygen (DO), specific conductance (Cond), turbidity (NUT), and pH using an Environmental Monitoring System probe (YSI EXO1, United States), and water transparency assessed with a Secchi disk (SD). In the laboratory, variables such as ammonium nitrogen (NH_4_-N), nitrate nitrogen (NO_3_-N), total nitrogen (TN), dissolved phosphate (PO_4_-P), total phosphorus (TP), and chemical oxygen demand (COD_Mn_) were measured using a segmented flow analyzer (Skalar San^++^, Netherlands; Cai, 2007).

Phytoplankton samples (1 L) were promptly preserved with 1.5% acidic Lugol’s solution and stored in darkness at 20°C until further processing. After 48 h of sedimentation, they were concentrated to 30 mL using a self-made siphon device. Identification of phytoplankton was carried out at 400× magnification using a light microscope. Taxa classification was performed following the methodology of Hu and Wei (2006). Bio-volume, representing the spatial occupation of individuals or group of cells in the water column, was estimated for each cell form using geometric formulae proposed by Hillebr et al. (2010). Volume measurements were then converted to biomass using assuming a conversion factor of 1 mm^3^ volume to 1 mg fresh-weight biomass.

### 2.3 Statistical analysis

The Trophic level index (TLI) was employed to evaluate the lake’s trophic status based on key water quality parameters, including TN, TP, COD_Mn_, Chl.*a* and SD (Wang et al., 2002).

The TLI was calculated using the following formula:


$$TLI\left(\sum \right)=\overset{m}{\underset{j=1}{\sum }}W_{j}\times TLI\left(j\right)$$

where *TLI (j)* is the nutritional status index of parameter *j* and *W_j_* is the relevant weight of the nutritional status index of parameter *j*.

The dominant phytoplankton taxa are identified based on their dominance values (Y), calculated as follows:


$$Y=\left(N_{i}/N\right)\times f_{i}$$

where *Ni* and *N* are the numbers of individuals of species *i* and the total number of individuals of all species within site, *fi* is the occurrence frequency of the species *i*. Species with Y values greater than or equal to 0.02 were categorized as dominant species (Ministry of Agriculture of the People’s Republic of China, 2010). The Shannon-Wiener diversity index (H′), Pielou evenness index (J), and Margalef richness index (d) were used to analyze diversity indices. The water body ecological status was evaluated using the Q index method. The Q index is calculated as follows:


$$Q=\overset{n}{\underset{i=1}{\sum }}\left[\frac{n_{i}}{N}\cdot F_{i}\right]$$

where *n* is the number of phytoplankton functional groups, where *n_i_* biomass of a *ith* functional group and *N* total biomass, and the factor *Fi* is the assigned value of the *ith* functional group. A Q index from 0 to 5 represents different water quality status: 0–1 is poor, 1–2 is tolerant, 2–3 is moderate, 3–4 is good, and 4–5 is very good (Padisák et al., 2006; Becker et al., 2010).

A detrended correspondence analysis (DCA) was conducted in advance to ascertain the suitability of redundant discriminant analysis (RDA) or canonical correspondence analysis for the given dataset. The maximum axis length across all DCA models was less than 4, suggesting that RDA was an appropriate method for analyzing both taxonomic diversity and trait composition for this study (Lai, 2013). Additionally, forward selection procedures and Monte Carlo permutations (499 iterations) were employed to identify those factors that exhibited a statistically significant influence (*p* < 0.05) on phytoplankton communities. The RDA was executed utilizing the CANOCO 5.0 (Microcomputer Power, New York, United States). Abiotic and biological data were log-transformed [log (x + 1)] for ordination analysis. The significance of environmental variables in explaining the variability of functional groups data in RDA was evaluated tested using Monte Carlo simulations with 499 permutations. Additional charts were generated using Origin Pro 2021 and Excel 2019.

## 3 Results

### 3.1 Variations of physical and chemical characteristics

The Daming Lake water exhibited a slightly alkaline pH characteristic, consistent with lakes receiving surface runoff. The TN exceeded V-class water standards, while the TP fell within the IV~V class range (GB3838-2002). COD_Mn_ levels in May were significantly higher than other periods, reaching 24.53 mg/L in 2021, with no significant difference compared to Xiaoqing River (unpublished data). The means of DO, TN, TP, and COD_Mn_ showed a decreasing trend from spring to autumn, whereas Sal, ORP, and SD displayed opposite trends. DO, TN, TP, and COD_Mn_ exhibited overall changes from spring to summer to autumn. Variance analysis results showed significant seasonal variations (*p* < 0.05) in pH, ORP, TP, and COD_Mn_, while other physicochemical factors did not show significant differences (*p* > 0.05; Table 1).

**Table 1:** Environmental factors during the sampling periods in the Daming Lake.

Parameters	Sampling periods					*p*-value
	May in 2020	July in 2020	November in 2020	May in 2021	September in 2021	
WT/°C	24.54 ± 0.44	27.52 ± 1.13	14.48 ± 0.55	22.92 ± 0.93	23.45 ± 0.46	0.30
DO/(mg/L)	12.83 ± 0.73	11.15 ± 1.5	10.29 ± 0.5	15.11 ± 1.07	11.76 ± 1.14	0.09
Sal/(mg/L)	0.40 ± 0.01	0.36 ± 0.04	0.44 ± 0.01	0.40 ± 0.01	0.42 ± 0	0.08
pH	8.05 ± 0.08	8.27 ± 0.22	7.86 ± 0.09	8.25 ± 0.25	7.68 ± 0.07	0.02^*^
ORP/mV	103.00 ± 7.79	254.33 ± 61.32	329.39 ± 13.59	808.50 ± 24.28	859.63 ± 10.56	0.001^**^
NO_3_^−^-N/(mg/L)	3.93 ± 0.81	2.34 ± 0.67	2.47 ± 0.56	3.18 ± 0.82	4.67 ± 1.06	0.49
NTU	7.20 ± 2.46	8.64 ± 0.98	5.24 ± 1.93	5.24 ± 2.01	5.80 ± 2.85	0.33
SD/m	0.66 ± 0.13	0.46 ± 0.05	1.05 ± 0.54	0.54 ± 0.07	0.59 ± 0.11	0.06
TN/(mg/L)	4.62 ± 0.82	2.79 ± 0.7	2.71 ± 0.47	6.46 ± 1.51	5.10 ± 0.83	0.21
TP/(mg/L)	0.07 ± 0.03	0.03 ± 0.01	0.03 ± 0.01	0.05 ± 0	0.04 ± 0.01	0.001^**^
COD_Mn_/(mg/L)	10.87 ± 2.78	7.54 ± 2.3	5.04 ± 4.72	24.35 ± 6.28	6.15 ± 1.09	0.004^**^
N:P	84.69 ± 50.64	99.24 ± 30.64	90.27 ± 37.57	132.22 ± 28.13	116.65 ± 24.08	0.24

The significance of the regression coefficients is indicated by **p<0.01; *p<0.05.

### 3.2 Phytoplankton community structure and functional group composition

#### 3.2.1 Phytoplankton community composition

**Figure 2:**
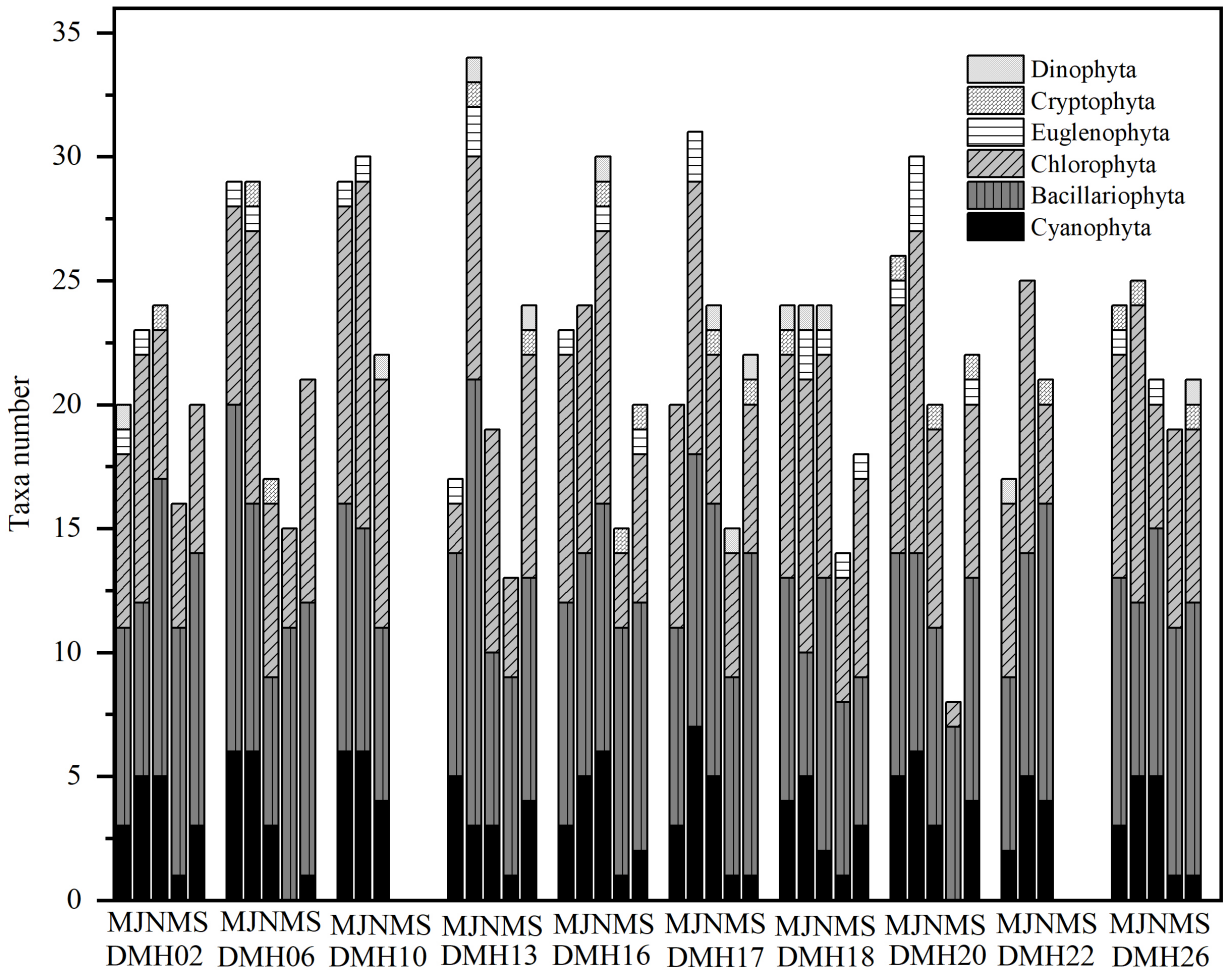
Composition of phytoplankton groups at various sampling periods in Daming Lake.

Throughout the survey period, a total of 98 phytoplankton taxa (including varieties and forma) belonging to 6 divisions (Cyanophyta, Chlorophyta, Bacillariophyta, Cryptophyta, Euglenophyta, and Dinophyta) were observed from Lake Daming, with Chlorophyta (39 species), Bacillariophyta (35 species), Cyanophyta (15 species), representing over 90.82% of the total phytoplankton community (Figure 2). Conversely, Euglenophyta, Dinophyta, and Cryptophyta exhibited minimal species diversity, accounting for only 9.18%. In terms of seasonal distribution, 72 species were recorded in the spring of 2020, with 31 species of Chlorophyta constituting 43% of the total phytoplankton; 82 species were identified in summer of 2020, including 34 species of Chlorophyta (42%); and 74 species were recorded in autumn of 2020, with 28 Bacillariophyta species (38%). In the spring of 2021, a total of 56 species were identified, including 22 species from the Bacillariophyta (30%), and 21 species from the Chlorophyta (28%); In September 2021, a total of 61 species from 6 phyla were identified, including 26 species of Bacillariophyta (35%), and 22 species from the Chlorophyta (30%).

#### 3.2.2 Density and biomass of phytoplankton

**Figure 3:**
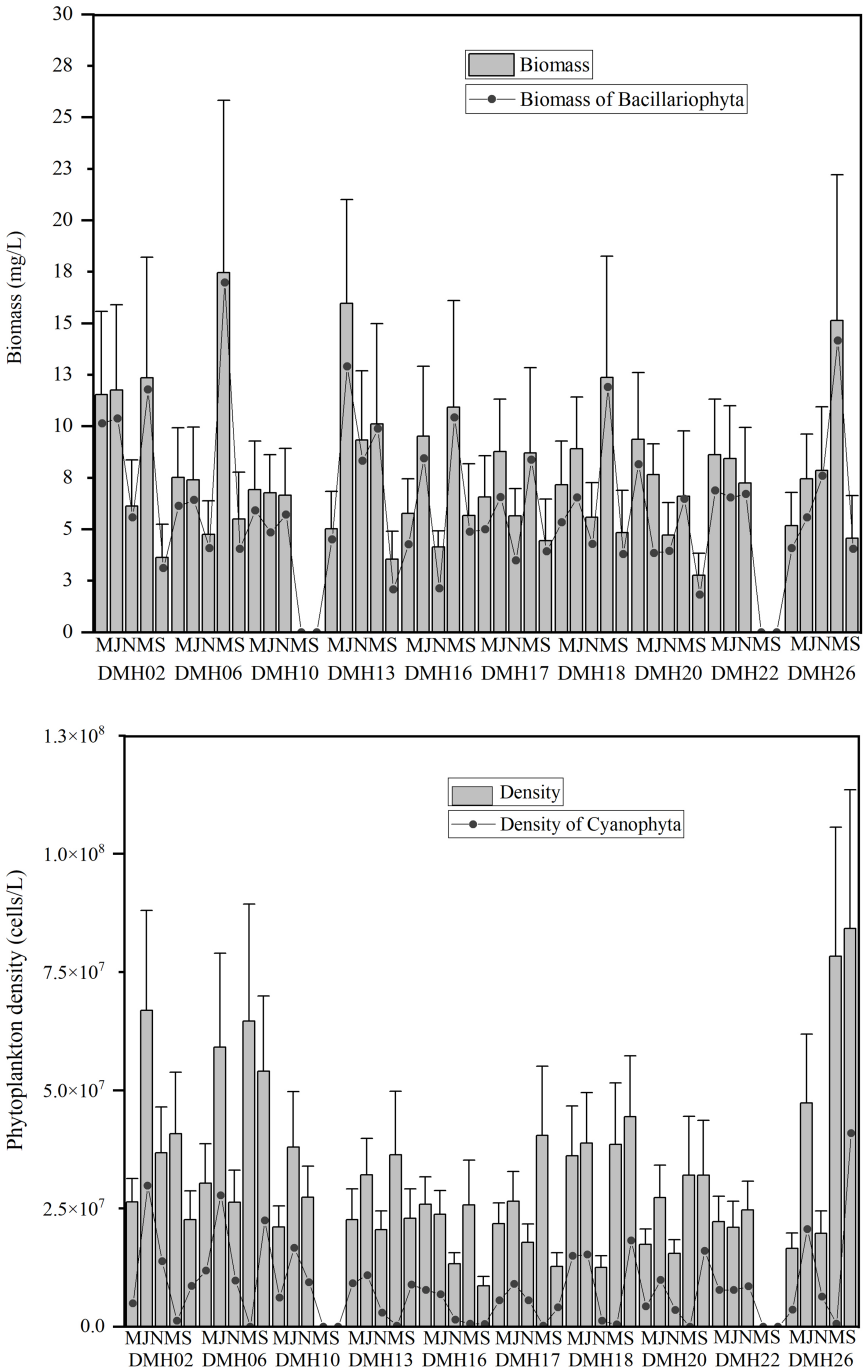
Phytoplankton density and biomass of Daming Lake in different sites and seasons.

As showed in Figure 3, the density and biomass of phytoplankton in Daming Lake exhibit a relatively consistent temporal variation. The phytoplankton density through the study varied between 0.97 × 10^7^ and 1.6 × 10^8^cells/L, with average densities of 2.4 × 10^7^cells/L, 3.8 × 10^7^cells/L, 2.1 × 10^7^cells/L, 4.5 × 10^7^ cells/L and 6.2 × 10^7^ cells/L, respectively. Cyanophyta dominates with peak density observed at DMH26 in September 2021. Regarding biomass, phytoplankton biomass ranges from 0.01 to17.46 mg/L, with average values of 1.71, 2.15, 1.44, 11.70, and 4.37 mg/L, respectively. Bacillariophyta prevails with the highest biomass at DMH06 in May 2021. No significant differences in biomass were observed among seasons at each site (*p* < 0.05).

The value of Y ranged from 0.02 to 0.72 during the 5 sampling periods, with *Microcystis* sp., *Merismopedia minima* and *Synedra acus* being identified as dominant species. And a total of 14 dominant species were found, including 7 species in May 2020, 5 species in July 2020, 6 species in November 2020, 5 species in May 2021, and 5 species in September 2021 (Table 2). Significant variations were noted in the distribution of dominant species, with Cyanophyta and Chlorophyta prevailing in May 2020 and September 2021, Cyanophyta in July 2020, and Bacillariophyta in November 2020 and May 2021.

**Table 2:** Dominant species of phytoplankton in Daming Lake during different sampling periods.

Dominant species		*Y*				
		May 2020	July 2020	November 2020	May 2021	September 2021
Cyanophyta	*Microcystis* sp.	0.37	0.20	0.02	--	--
	*Merismopedia minima*	0.72	0.42	0.31	--	--
	*Merismopedia sinica*	--	0.04	--	--	--
	*Merismopedia tenuissima*	--	--	--	--	0.65
	*Phormidium tenus*	--	--	0.03	--	--
Bacillariophyta	*Cyclotella* sp.	0.02	0.03	--	--	0.02
	*Melosira granulata*	--	--	0.13	--	--
	*Stauroneis anceps*	--	--	--	0.08	0.02
	*Nitzschia amphibia*	--	--	--	0.12	--
	*Achnanthes catenata*	--	--	--	0.56	--
	*Synedra acus*	0.18	0.07	0.05	0.05	0.03
Chlorophyta	*Scenedesmus quadricauda*	0.15	--	0.04	0.04	0.04
	*Scenedesmus dimorphus*	0.02	--	--	--	--
	*Crucigenia tetrapedia*	0.02	--	--	--	--

#### 3.2.3 Classification of functional groups of phytoplankton

These 98 phytoplankton taxa were classified into 21 functional groups: B, C, D, F, G, H1, J, K, Lo, M, MP, N, P, S1, S2, S_N_, W1, X1, X2, X3, and Y (Table 3). Among these, the J functional group exhibited the highest species richness with 24 species, predominantly including *Scenedesmus*, *Pediastrum*, *Crucigenia*, and *Tetraedron*. The MP functional group was characterized by *Surirella*, *Cymhella*, and *Achnanthes*; the D functional group primarily consisted of *Synedra* and *Nitzschia*; the Lo functional group was dominated by *Merismopedia* and *Peridinium*; the P functional group was mainly composed of *Melosira* and *Fragilaria*.

**Table 3:** Main phytoplankton taxonomic and functional groups in the Daming Lake.

Functional groups	The most abundant representatives	Taxonomic group	The corresponding habitats	F
B	*Cyclotella* sp., *Stephanodisus neoastraea*	Bacillariophyta	Vertically mixed, mesotrophic small-medium lakes with species sensitive to the onset of stratification	4
C	*Asterionella formosa*	Bacillariophyta	Mixed, eutrophic small medium lakes	2
D	*Synedra acus*, *Synedra ulna*	Bacillariophyta	Shallow, enriched turbid waters, including rivers	2
F	*Dictyosphaerium pulchellum*, *Oocystis lacustris*	Chlorophyta	Clear epilimnia	5
G	*Pandorina morum*	Chlorophyta	Short, nutrient rich water	2
H1	*Anabaena oscillarioides*, *Anabaena circinalis*	Cyanophyta	Eutrophic, both stratified and shallow lakes	0
J	*Scenedesmus quadricauda*, *Actinastrum hantzschii*	Chlorophyta	Shallow, enriched lakes ponds and rivers	2
K	*Aphanocapsa pulchra*	Cyanophyta	Short, nutrient-rich columns	2
Lo	*Merismopedia tenuissima*, *Peridinium bipes*	Cyanophyta, Pyrrophyta	Deep and shallow, oligo to eutrophic lakes	2
M	*Microcystis* sp.	Cyanophyta	Dielly mixed layers of small eutrophic, low latitude lakes	0
MP	*Cymhella affinis*, *Surirella capronii*, *Achnanthes catenata*	Bacillariophyta	Frequently stirred up, turbid shallow lakes	4
N	*Tabellaria fenestrata*, *Cosmarium circulare*	Chlorophyta	Continuous or semi-continuous mixed layer	3
P	*Melosira granulata*, *Melosira varians*	Bacillariophyta	Eutrophic epilimnia	2
S1	*Phormidium tenue*, *Lyngbya limnetica*	Cyanophyta	Turbid mixed layers	0
S2	*Arthrospira platensis*	Cyanophyta	Warm, shallow and often highly alkaline waters	3
S_N_	*Raphidiopsis curvata*	Cyanophyta	Warm mixed layers	0
W1	*Euglena acus*, *Phacus acuminatus*	Euglenophyta	small organic ponds	2
X1	*Chlorella* sp., *Ankistrodesmus acicularis*	Chlorophyta	Shallow, eu-hypertrophic environments	3
X2	*Chlamydomonas simplex*, *Chroomonas acuta*	Chlorophyta, Cryptophyta	Shallow, meso-eutrophic lakes	3.5
X3	*Cymatopleura solea*, *Gyrosigma acuminatum*	Bacillariophyta	Shallow, well mixed oligotrophic layers	4
Y	*Cryptomonas ovata*, *Glenodinium* sp.	Cryptophyta	small, enriched lakes	3

**Figure 4:**
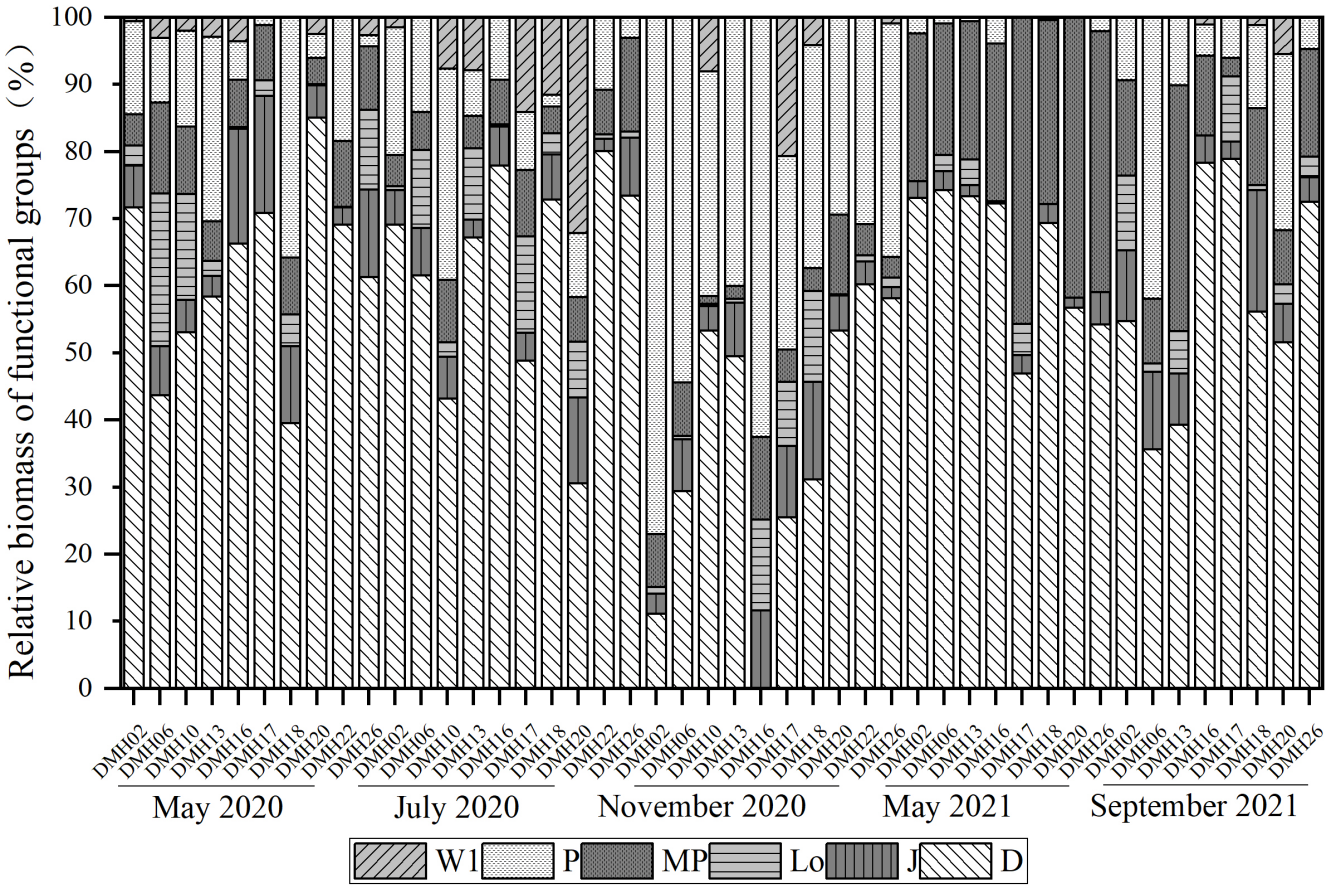
Variation characteristics of dominant functional groups in different periods of Lake Daming.

The identified phytoplankton species were classified into 21 functional groups, among which 6 dominant groups (relative biomass >5%) were identified, namely, D, P, J, MP, W1 and Lo (Figure 4). Of the 19 FGs identified in the lake, 5 were dominant groups, namely, D, P, J, MP and Lo with the mean relative biomass were 56.81, 12.06, 7.58, 7.07, and 5.38% in spring 2020, respectively. In July 2020, there were 18 functional groups, 5 of which [i.e., D (57.12%), P (9.81%), MP (6.37%), W1 (6.63%), Lo (5.62%), and J (5.12%)] were dominant. Functional groups D, P and J had the greatest mean relative biomasses at 38.06%, 37.98%, and 6.27% in November 2020, respectively (Figure 4). The relative biomass of the dominant groups in May 2021 were markedly different from those in spring and summer, with group D (65.31%) and group MP (28.63%) being the most dominant. In September 2021, the dominant functional groups of phytoplankton were D (55.26%), P (12.86%), MP (11.87%), and J (7.60%). The composition of phytoplankton functional groups varies significantly between different seasons. The D functional group thrives in water bodies with high turbidity and nutritional index, indicating a eutrophic tendency in the water quality of Daming Lake.

#### 3.2.4 Characteristics of the dominant functional groups

Based on the variations in dominant taxa across different seasons and sites, it is evident that the D functional group, characterized by species of *Synedra*, exhibited the highest frequency and dominance in all seasons, with its biomass significantly surpassing that of other functional groups. The P functional group, represented by species of *Melosira*, and the MP functional group, represented by species of *Achnanthes catenata*, followed as the next dominant functional groups. In contrast, the J, Lo and W1 functional groups were the least dominant, displaying the lowest occurrence frequency. The J functional group, predominantly comprising species of *Scenedesmus* and the Lo functional group, represented by species of *Merismopedia*, were primarily observed in spring and summer, while the W1 functional group, represented by species of *Euglena*, was only present in summer. Thus, the D functional group emerged as the unequivocal dominant group of phytoplankton in Daming Lake, with the P, MP, J, Lo and W1 functional groups serving as the secondary dominant. The overall succession pattern of phytoplankton functional groups in Daming Lake was D/P/J/MP/Lo → D/P/W1/MP/Lo/J → D/P/J → D/MP → D/P/MP.

### 3.3 RDA analysis of functional groups of phytoplankton and environmental factors

**Figure 5:**
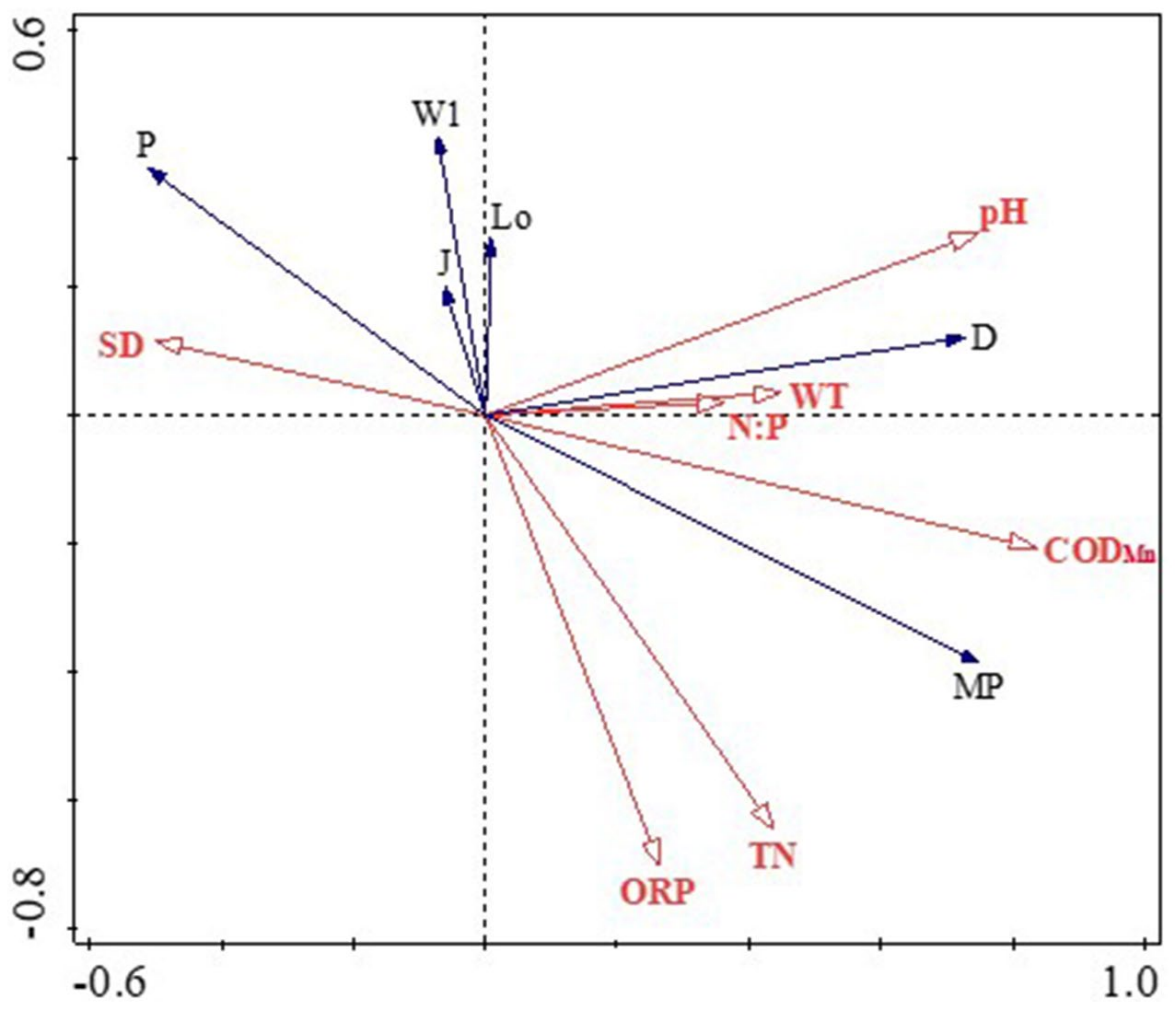
RDA analysis of dominant functional groups and environmental factors in Lake Daming.

The phytoplankton functional group detrended correspondence analysis (DCA) eigenvalue in this study was below 2, validating the suitability of a linear model and prompting the application of RDA for further analysis (Lai, 2013). Eigenvalues for the first and second axes of 0.4836 and 0.0524, respectively (Figure 5), collectively explaining 53.59% of the cumulative variance in dominant functional group biomass and elucidating the association between functional groups and environmental factors (axis 1: 48.36%; axis 2: 5.24%). RDA results depict that phytoplankton biomass and composition were driven more by the availability of nutrients such as WT, pH, ORP, SD, COD_Mn_, TN, and N:P in Lake Daming. Specifically, dominant functional groups D and MP exhibited positive correlations with WT, pH, N:P and COD_Mn_, while displaying negative correlations with SD. Additionally, other major functional groups that contributed to phytoplankton community variations showed significant correlations with environmental variables, such as functional groups W1, J, Lo and P were significantly positively correlated with SD, while negatively associated with TN, ORP and COD_Mn_, which corroborated the RDA results (Figure 5).

### 3.4 Comprehensive evaluation of aquatic ecology based on phytoplankton

#### 3.4.1 Evaluation of trophic level index

The trophic level index (TLI) of Daming Lake was calculated using chlorophyll a (Chl a), Secchi disk depth (SD), TN, TP, and COD_Mn_ as parameters. Analysis revealed that the TLI ranged from 39.10 to 71.13 across all sampling points, with an average value of 58.70. Assessment based on TN and TP nutritional status indices classified the water body in Daming Lake as mildly eutrophic according to lake nutritional status classification (Wang et al., 2002).

#### 3.4.2 Evaluation of phytoplankton diversity index

**Figure 6:**
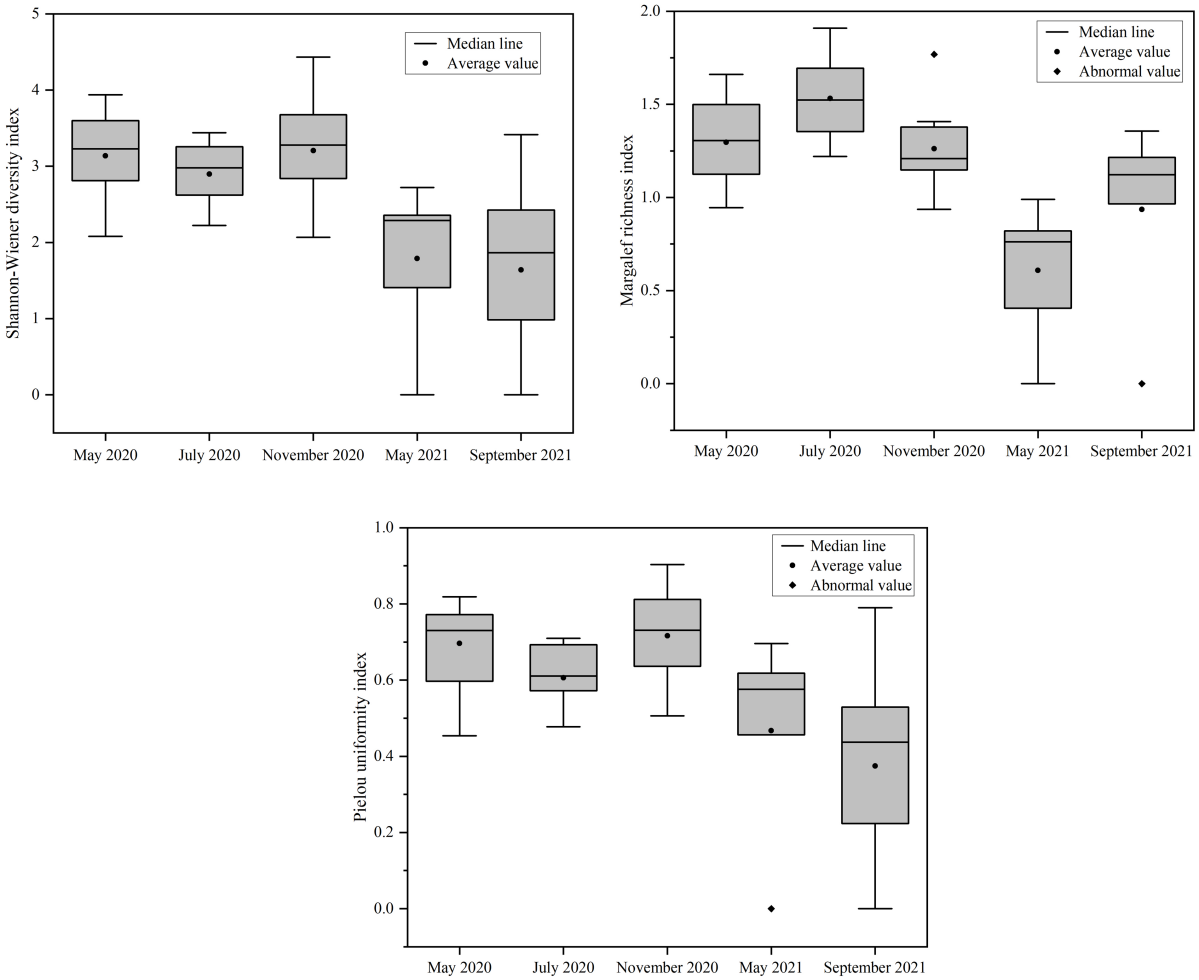
Variation of phytoplankton species diversity in Lake Daming in different sampling periods.

The statistical evaluation of phytoplankton diversity indexes in Daming Lake across different seasons is illustrated in Figure 6. The results indicate that there is no significant difference in the Shannon-Wiener diversity index H′ between the various seasons (*p* > 0.05). However, the J value of phytoplankton was higher in 2020 compared to 2021, and the *d* value in July 2020 was notably higher than during other periods. Overall, it can be inferred that the water quality of Daming Lake within a range from mild to moderate pollution levels.

#### 3.4.3 Evaluation of ecological status (Q index)

**Figure 7:**
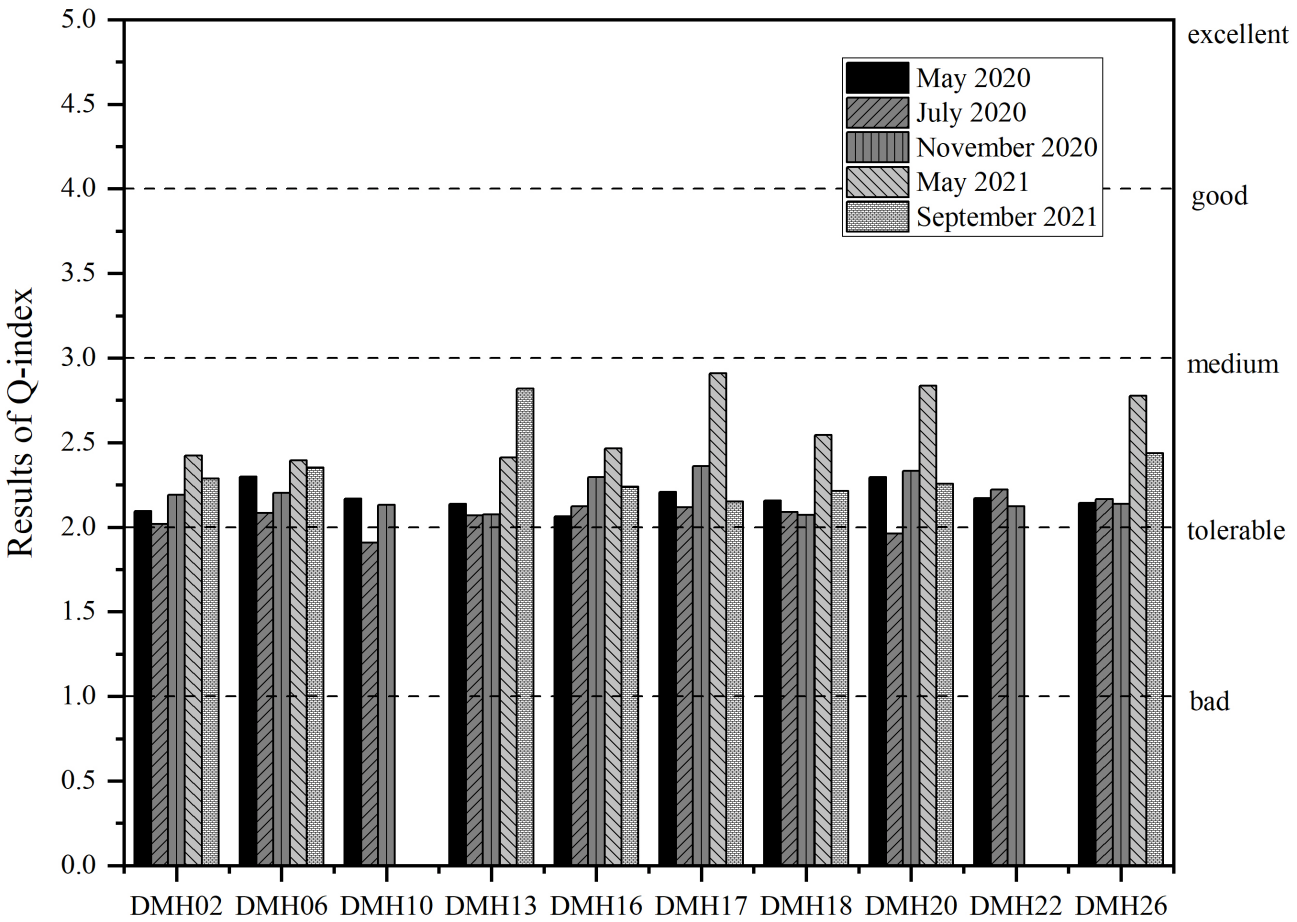
Q index of phytoplankton functional groups at each site in different sampling periods.

Based on the habitat variances in Daming Lake area, distinct F factors were allocated to each functional group for the calculation of the Q index (Figure 7). Analysis revealed that the Q index ranged from 1.91 to 2.91 across three sampling periods in Daming Lake, indicating a generally “moderate” ecological health status.

## 4 Discussion

### 4.1 Water quality and phytoplankton community characteristics

The findings of a survey conducted in 2021 revealed that during the summer and autumn was approximately 19.26°C, which is significantly lower than the lake’s own temperature of around 23.18°C. The nutrient levels of nitrogen and phosphorus in Daming Lake, both lower than those of Baiyun Lake and Xiaoqing River in Jinan (unpublished data), are comparable to those found in other urban lakes in China such as Wuhan and Nanjing (Zhang et al., 2023; Qu and Zhou, 2024). A total of 6 phyla and 98 taxa (including varieties and forms) of phytoplankton were identified in Daming Lake, with an average density of 3.67 × 10^7^ cells/L from 2020 to 2021. However, no significant differences were observed regarding phosphorus levels. Additionally, it was noted that the average phytoplankton density in rivers entering the lake was only 5.1 × 10^6^ cells/L, much lower than the density in Daming Lake (3.99 × 10^7^ cells/L). The species diversity and density post-dredging and cleaning were significantly higher. Chlorophyta and Bacillariophyta were the dominant groups in Daming Lake, with *Synedra acus*, *Cyclotella* sp., *Scenedesmus quadricauda*, *Merismopedia minima* and *Microcystis* sp. as the prevailing species throughout the entire investigation period. Cyanophyta exhibited high density, while diatoms had a substantial biomass proportion. The findings are in line with previous studies, indicating a predominance of Chlorophyta, Bacillariophyta and Cyanophyta (Zheng et al., 2017; Lu et al., 2019). The species composition and dominant species align with those observed in urban lakes influenced by surface runoff, such as Wuhan, Portland, and Bucharest (Florescu et al., 2022; Popa et al., 2023; Yan et al., 2023). Phytoplankton functional groups, categorized based on adaptability and habitat conditions, offer a more precise depiction of the functional traits and dynamic interrelations within phytoplankton communities and their aquatic environment (Yan et al., 2023). Identified phytoplankton were classified into 21 functional groups, including 19 in spring 2020, 18 in the summer and autumn of 2020, 14 in spring and autumn of 2021. Key functional groups in 2020 include D, P, J, Lo and MP while those in 2021 consist of D, MP, J and P. Functional group D (primarily *Synedra*) and B (mainly *Cyclotella*) have dominated Daming Lake, but an increasing prevalence of functional group S_N_ and emergence of Cyanophyta-dominant groups like TC, M, and Lo have been observed since 2014 (Table 4).

**Table 4:** Phytoplankton community succession in Daming Lake from 2010 to 2021.

Time (year)	2010 (Wang et al., 2022)	2011 (Wang et al., 2022)	2012 (Wang et al., 2022)	2013 (Wang et al., 2022)	2014 (Wang et al., 2022)	2015 (Lu et al., 2019)	2017 (Li et al., 2023)	2020	2021
Dominant taxa	*Synedra*, *Cyclotella*	*Synedra*, *Cyclotella*, *Raphidiopsis*	*Synedra*, *Cryptomonas*	*Synedra*, *Fragilaria*	*Synedra*, *Oscillatoria*	*Synedra*, *Cyclotella, Oscillatoria*, *Scenedesmus*	*Phormidium*, *Raphidiopsis*	*Microcystis*, *Merismopedia*, *Synedra*, *Cyclotella*	*Synedra*, *Stauroneis*, *Achnanthes*
Dominant FGs	B, D	B, D, S_N_	D, X2	D, P	D, TC	B, D, J, TC	TC, S_N_	D, P, J, MP, Lo	D, MP, J, P
Density(10^5^cells/L)	1.88	2.18	1.2	0.56	0.09	0.36	224.00	378.33	534.02

### 4.2 Phytoplankton functional groups response to physical–chemical variables

Variations in physicochemical elements often affect the composition and structure of phytoplankton in aquatic ecosystems. Consistent with previous multiple researches on other urban lakes have indicated that the primary factors influencing the composition of phytoplankton communities in various aquatic environments differ significantly with key factors being water WT, TN, SD, N:P, ORP, COD_Mn_ and pH (Zhang et al., 2023; Etisa et al., 2024; Wu et al., 2024). Enzymatic activity within cells, which influences algae metabolism, is predominantly affected by temperature, making it a crucial factor in phytoplankton distribution (Su et al., 2007; Krashchuk et al., 2020). Compared with other phyla, diatoms thrive in cooler temperatures, with an optimal growth temperature of 20°C. The dominant functional groups D (represented by *Synedra acus, Synedra ulna*) and MP (including *Cyclotella* sp. *Cymhella affinis*, *Surirella capronii*) in Daming Lake are able to adapt to turbid shallow environments with frequent disturbances and high tolerance to lower temperatures (Padisák et al., 2009; Etisa et al., 2024). The main water supply of Daming Lake comes from springs with a significantly lower water temperature than that of the lake, resulting in low temperatures and high turbidity. Additionally, due to the shallow landscape nature and the temperate monsoon climate of the lake, water mixing occurs frequently, prolonged convective rainstorms leading to substantial precipitation. Consequently, the lake receives a high influx of exogenous pollutants, leading to decreased water transparency and increased nitrogen and phosphorus nutrient loads, which promote the growth of dominant functional groups such as D and MP. Consistent with the correlation, the RDA results revealed that the two groups were well correlated with the concentration of TN, N:P, COD_Mn_ and SD in water (Figure 5). This result is inconsistent with those of many studies that suggest phosphorus as the limiting nutrient for phytoplankton in different water bodies (Cao et al., 2018; Ding et al., 2022). In fact, during the study period, the average TN concentrations in Daming Lake ranged from 2.71 mg/L to 6.46 mg/L. The P group primarily thrives in moderate to eutrophic, persistent or semi-persistent mixed water bodies, and is tolerant of low light intensity. The P function group is widely distributed in Daming Lake, with its density accounting for up to 37.9% in autumn. This is consistent with the low water level and transparency of Daming Lake, providing suitable habitat conditions for the growth of the P group represented by *Melosira granulata*. COD_Mn_ is a comprehensive index reflecting the pollution level of organic pollutants and inorganic reducing substances in water bodies, which is positively correlated with the growth of phytoplankton (Han et al., 2022; Yan et al., 2023). The extensive growth of D, MP and other functional groups may be a significant factor contributing to the increase of COD_Mn_. These findings indicated that the habitat characteristics of phytoplankton functional groups align well with the environmental characteristics of Daming Lake. The use of functional groups as a method can effectively identify water environmental characteristics.

**Figure 8:**
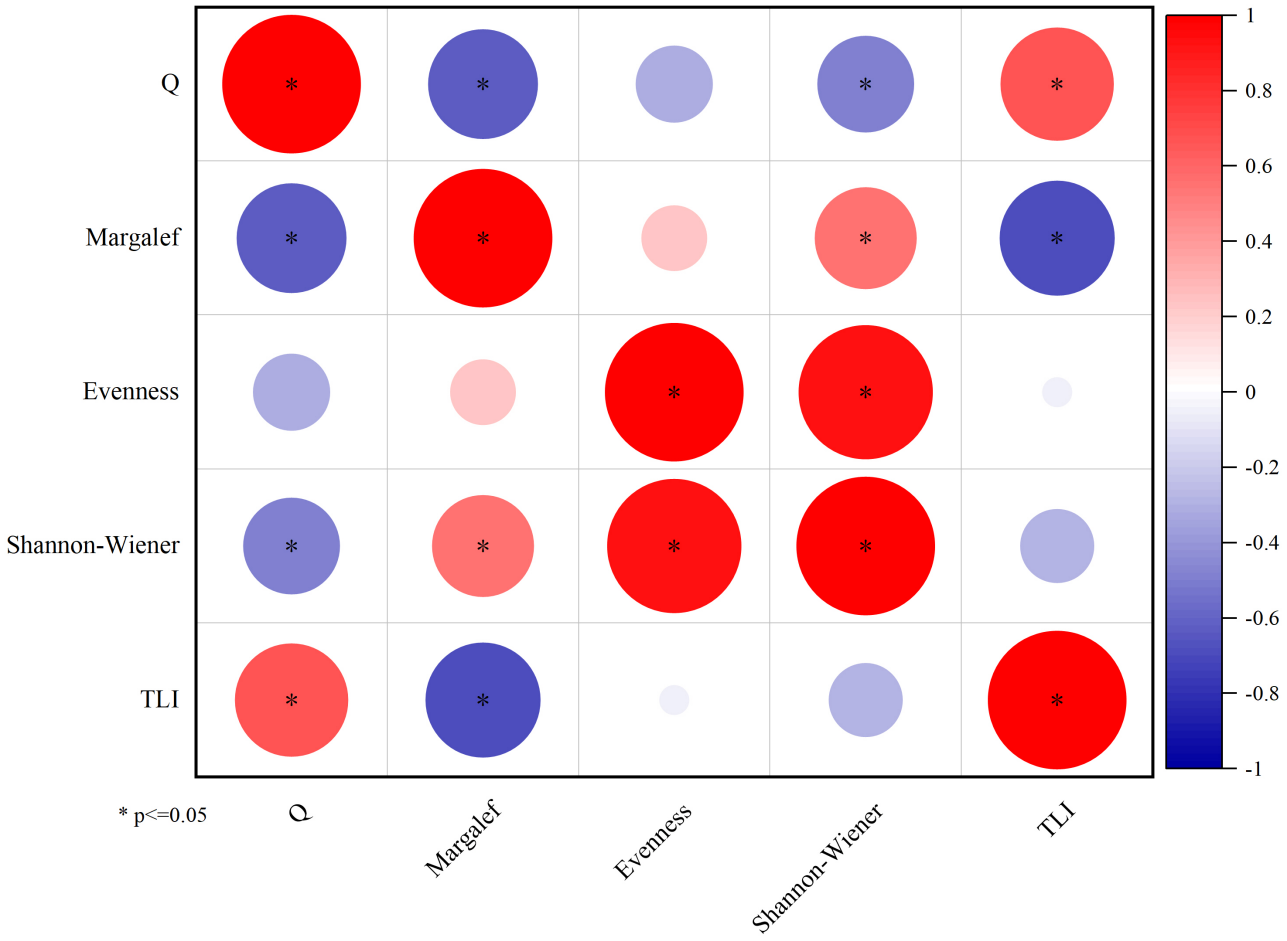
The correlation between Q, diversity index and TLI index.

This study employs the Shannon-Wiener index and TLI, Q index based on functional groups to comprehensively assess the water quality of Daming Lake. The findings reveal that Q index have a significant correlation with Shannon-Wiener, Marglef index and TLI (*p* < 0.05), and the overall assessment grade derived from these three indicators remains consistent (Figure 8). The evaluation indicates that Daming Lake was in a state of moderate eutrophication, demonstrating the suitability of the functional group-based evaluation system for assessing water quality of Daming Lake.

## 5 Conclusion

1. As a spring-fed lake, the phytoplankton community structure of Daming Lake exhibited similarities to other urban lakes, with 98 taxa identified across 6 phyla, encompassing 21 functional groups. Cyanophyta and Bacillariophyta were the primary biomass contributors, especially Bacillariophyta with its significant biomass proportion. Functional groups D, P and MP were identified as the most important groups in terms of functional group composition.
2. RDA analysis indicated that WT, TN, SD, N:P, ORP, COD_Mn_ and pH were the key environmental factors influencing the FGs of Daming Lake.
3. The trophic level index, diversity index, and ecological status index collectively suggested that the water quality of Daming Lake is at a moderate health level. The Q index, established on functional group categorization, is deemed appropriate for assessing the water environmental quality of Daming Lake.

## Data availability statement

All relevant data is contained within the article: The original contributions presented in the study are included in the article/supplementary material, further inquiries can be directed to the corresponding author/s.

## Author contributions

HS: Data curation, Funding acquisition, Investigation, Writing – original draft, Writing – review & editing. HX: Data curation, Formal analysis, Software, Writing – review & editing. XZ: Software, Writing – review & editing. TZ: Data curation, Funding acquisition, Investigation, Writing – review & editing. WJ: Data curation, Investigation, Writing – review & editing. XL: Funding acquisition, Investigation, Resources, Software, Writing – review & editing.
